# The Production of Asthenia or Anæsthesia in Surgical Operations by Compression of the Vagus Nerves

**Published:** 1871-04

**Authors:** 


### THE PRODUCTION OF ASTHENIA OR ANÆSTHESIA IN SURGICAL OPERATIONS BY COMPRESSION OF THE VAGUS NERVE.—(*The Practitioner*, December, 1870.)

In a short paper, probably the last ever written by its distinguished author, the late Dr. Augustus Waller, of Geneva, shows that pressure exerted upon the vagus nerve in the neck produces on man most of the principal symptoms observed on animals when that nerve is isolated and subjected to the direct influence of galvanism. Upwards of twenty years ago, when studying the subject of compression with reference to hysteria and epilepsy, two cases occurred where compression of the vagus was followed by all the symptoms described by Aristotle "In each case the patient, after moderate pressure, fell down, as if struck by lightning, on the floor before me, like a lifeless corpse, with all the voluntary muscles relaxed. Scarcely had I time to become alarmed, when sensation and voluntary power returned, although for some time afterwards there remained considerable weakness and debility, though not sufficient to prevent the patient from walking away unassisted."

These symptoms appear to the ordinary observer to be attended with considerable danger, but such, in reality, is not the fact, as the heart is always found to be pulsating, and the respiration in play. He therefore recommends its adoption in cases where the abolition of muscular power may be desirable, as in instances of dislocated bones of difficult reduction, previous to the employment of the ordinary anæsthetics, the administration of which is attended with a certain amount of danger. As an illustration of the practice, he narrates the case of a powerful, athletic man, in whom, in consequence of a fall, the head of the humerus was dislocated beneath the clavicle. An ineffectual effort had been made to reduce it without chloroform, and, while the messenger was procuring that agent, compression was made of both vagi, while extension and counter-extension were kept up. At the end of two or three minutes, just as the two carotids could no longer be felt beating beneath the fingers, a sudden click indicated the return of the bone into its socket.

As an illustration of the anæsthetic effects of vagal pressure, he says, "A molar tooth was extracted from an out-patient of the Hôpital Cantonel by one of the house surgeons. While the patient was seated, I was prepared, at the back of the chair, to apply pressure on both vagi. As soon as the key was gently applied around the tooth, I began the pressure, and gave a sign to the operator to commence. The result was perfectly satisfactory. According to the statement of the patient, she had suffered no pain, and was most enthusiastic in her thanks to me. At the moment of extraction the patient cried out, which, however, occurs in many instances with chloroform, where, as in this case, the patients afterwards declare they have not felt any pain."

